# Isolating a cerebellar contribution to rapid visual attention using transcranial magnetic stimulation

**DOI:** 10.3389/fnbeh.2012.00055

**Published:** 2012-08-24

**Authors:** Carla P. Arasanz, W. Richard Staines, Tom A. Schweizer

**Affiliations:** ^1^Department of Kinesiology, University of WaterlooWaterloo, ON, Canada; ^2^Faculty of Medicine, Division of Neurosurgery, University of TorontoToronto, ON, Canada; ^3^Keenan Research Centre of the Li Ka Shing Knowledge Institute at St. Michael's HospitalToronto, ON, Canada; ^4^Division of Neurosurgery, St. Michael's HospitalToronto, ON, Canada

**Keywords:** cerebellum, cognition, visual attention, theta burst stimulation, attentional blink

## Abstract

Patient and neuroimaging research have provided increasing support for a role of the posterior-lateral cerebellum in cognition, particularly attention. During rapid serial visual presentation, when two targets are presented in close temporal proximity (<500 ms), accuracy at detecting the second target (T2) suffers. This phenomenon is known as the attentional blink (AB), and in cerebellar lesion patients this effect is exaggerated. Damage to the cerebellum may thus disrupt the use of attentional resources during stimulus processing conditions that are temporally demanding. There are reciprocal connections between the cerebral cortex and the contralateral cerebellum, these connections allow for the possibility that lateralized functions in the cerebral cortex (such as language) remain lateralized in the cerebellum. The purpose of this study was to investigate the temporal characteristics of the cerebellar contribution to the AB and to functionally localize the contribution of the cerebellum to the AB using transcranial magnetic stimulation (TMS). We hypothesized that T2 accuracy would decrease after right cerebellar stimulation when the delay between the first target (T1) and T2 was short (120–400 ms) compared to long (720–960 ms). We used continuous theta burst stimulation (cTBS), a form of TMS, to transiently inhibit a focal population of neurons in the left and right posterior-lateral cerebellum of healthy participants (*n* = 45). Three groups of participants (*n* = 15) performed the AB before and after either sham, left, or right cerebellar stimulation. The results of this cTBS study support our hypothesis. During the short delay, participants in the right cTBS group showed a greater AB magnitude compared to both the left and sham cTBS groups (*p* < 0.05). No difference in T2 detection was found over long delays. The results provide further support for a cerebellar contribution to an integrated neural network recruited during temporally demanding attention-based tasks.

## Introduction

The attentional blink (AB), coined by Raymond et al. (1992), is a phenomenon that occurs when two targets are presented in rapid succession (200–500 ms) and the accuracy of detecting the second target (T2) is impaired at the cost of detecting the first (Broadbent and Broadbent, 1987; Raymond et al., 1992). There are many theoretical accounts for this phenomenon (for review see Dux and Marois, 2009); a common claim is that if two targets that require attention are presented too closely together, attending to the first target (T1) can delay the processing of the T2. This leaves T2 susceptible to interference and increases the chance of it going undetected. If, however, the stimulus onset asynchrony (SOA) between T1 and T2 is long, T1 is processed before the presentation of T2, and accuracy is high for both targets. Thus, the deterioration of T2 accuracy when the SOA is short is the result of interference that occurs between stimuli during preliminary conceptual processing. At this stage, stimuli are vulnerable to being overwritten by subsequent stimuli. In order for a target to be encoded, it must enter a second stage of processing so that it can be consolidated into working memory. This stage, however, is capacity-limited, and consequently when T2 is presented in close temporal proximity to T1, it must wait to be encoded until T1 consolidation into working memory is complete (Chun and Potter, 1995; Vogel et al., 1998).

In support of this claim, recent neuroimaging studies have found that the magnitude of the AB is predicted by how much an individual devotes their attentional resources to T1 processing (Shapiro et al., 2006). A number of AB studies have used event-related potentials (ERPs) to target the amplitude and latency of the P300 component, which is characterized by a positive deflection distributed over the scalp with a latency of 300–500 ms. It is proposed that the P300 is related to post-perceptual processing, such as the updating of working memory and the conscious report of a target stimulus (Sergent et al., 2005; Del Cul et al., 2007). Kranczioch and colleagues (2007) found an inverse relationship between the P300 amplitudes time locked to T1 and T2, such that when T1's P300 was bigger, T2's P300 was smaller. This suggests that the more attention allocated to T1, the larger its neural response, and the less attentional resources are available for the processing of T2. Furthermore, an fMRI study of the AB that activated specific brain areas for T1 and T2 stimuli found that the level of activity in T1 visual object-encoding areas predicted detection of T2 (Slagter et al., 2010). These observations suggest that the AB does not necessarily reflect a bottleneck in information processing, but rather a processing strategy for how attentional resources are managed and allocated (Hommel et al., 2006).

Recent brain imaging and clinical studies have implicated a network of lateral frontal and posterior parietal areas involved in the conscious detection of targets in the AB. Functional MRI studies have shown greater activity in this network when T2 is detected compared to when T2 is missed, suggesting a highly distributed network is involved in attentional control (Marois et al., 2004; Kranczioch et al., 2005). The cerebellum, for example, forms a network with the lateral prefrontal cortex (Middleton and Strick, 2001; Schmahmann et al., 2004; Allen et al., 2005) and its activation has been associated with the AB (Marcantoni et al., 2003; Slagter et al., 2010; Hesselmann et al., 2011). Clinical lesion studies have also provided support for a cerebellar contribution in the AB (Schweizer et al., 2007). In this study, patients with focal cerebellar lesions performed equivalently to controls when detecting T1, and the duration of the AB effect was the same. There was, however, an increased AB magnitude specific to short SOAs, when T2 occurred within 500 ms of T1. This data provides evidence supporting the cerebellum as a critical node in the AB network.

For decades cerebellar patient studies have been documenting impairments that extend beyond the motor domain. Damage to the posterior-lateral cerebellum can result in purely cognitive deficits, such as those seen after lesions to prefrontal areas (Schweizer et al., 2008). Contralateral connections between the prefrontal cortex and the cerebellum allow for the possibility that lateralized functions in the cerebral cortex remain lateralized in the cerebellum. Language, for example, is heavily lateralized to the left cerebral cortex, and lesions to the right cerebellar hemisphere are associated with deficits in word generation tasks (Akshoomoff et al., 1992; Appollonio et al., 1993; Silveri et al., 1994; Molinari et al., 1997; Leggio et al., 2000; Richter et al., 2007; Schweizer et al., 2010) and verbal working memory (Hokkanen et al., 2006). AB paradigms predominantly use letter stimuli; it is therefore possible that the contribution of the cerebellum is right hemisphere specific.

The pattern of connectivity between the cerebellum and the contralateral cerebral cortex can be better understood using transcranial magnetic stimulation (TMS). Repetitive TMS (rTMS) delivers trains of stimuli at different frequencies and has been shown to disrupt function of cerebellar circuits during cognitive tasks (Oliveri et al., 2007). The strength of rTMS is that it is a technique that can transiently alter the function of the brain region directly targeted and can effectively change the activity of an associated distributed network (Mottaghy et al., 2002). In a previous study we used continuous theta burst stimulation (cTBS), a form of rTMS, to investigate hemispheric specificity of the cerebellum during word generation tasks. We found that cTBS to the right posterior-lateral cerebellum decreased performance during a word generation task, specifically during the early phase of the task, by diminishing the ability to efficiently organize word output (Arasanz et al., 2012). Our previous finding is the first evidence that the effects of cTBS on word generation are lateralized to the right cerebellar hemisphere and supports patient and imaging data for the role of the cerebellum in non-motor behavioral tasks, specifically when time is a constraint.

The purpose of this study is to investigate the temporal characteristics of the cerebellar contribution to the AB and to functionally localize the contribution of the cerebellum to the AB using cTBS. We hypothesize that T2 accuracy will decrease after right cerebellar stimulation and have no effect after left cerebellar and sham stimulation, when the delay between the T1 and T2 is short (120–480 ms) compared to long (720–960 ms).

## Materials and methods

### Participants

Forty-five healthy, right-handed participants (age range 20–35 years, mean = 23.3) with no reported history of neurological problems were recruited for this study. Participants were randomly assigned to one of three groups; Left, Right, or Sham stimulation of cTBS to the posterior-lateral cerebellum. There were 15 participants in each group. All participants provided written informed consent prior to testing. Experimental procedures were approved by the Office of Research Ethics at the University of Waterloo.

### Experimental task and stimuli

Participants were seated in a sound attenuating booth (Industrial Acoustics, 120A, NY), facing a computer screen at a viewing distance of 30 cm. Using EPrime software (Psychology Software Tools Inc, USA) stimuli were presented in black on a grey background as uppercase letters (9.1 cd/m^2^), which subtended a visual angle of 16.3° by 12.5°. Letters were presented in rapid serial visual presentation (RSVP; 120 ms/letter) where each letter appeared for 120 ms with no blank inter-stimulus interval (ISI). Within each trial two targets were embedded among a string of distractors. The T1 was either a white H or S and the T2 was a black X or Y. No letter was ever repeated within the letter stream and distractors were any letter of the alphabet excluding defined target letters. T1 occurred 7–15 letters after the central fixation cue. T2 was always one of eight letters that followed T1. T2 occurred with no (lag 1), one (lag 2), two (lag 3), three (lag 4), five (lag 6), or seven (lag 8) distractors after T1. Lags 1–4 were short lags occurring within 480 ms of T1, and lags 6 and 8 were classified as long lags occurring at least 720 ms after T1. A distractor replaced T1 on trials where no T1 was presented. This occurred for approximately one-third of all trials and served as a control condition where no AB effect should be present.

### Procedure

**Figure 1:**
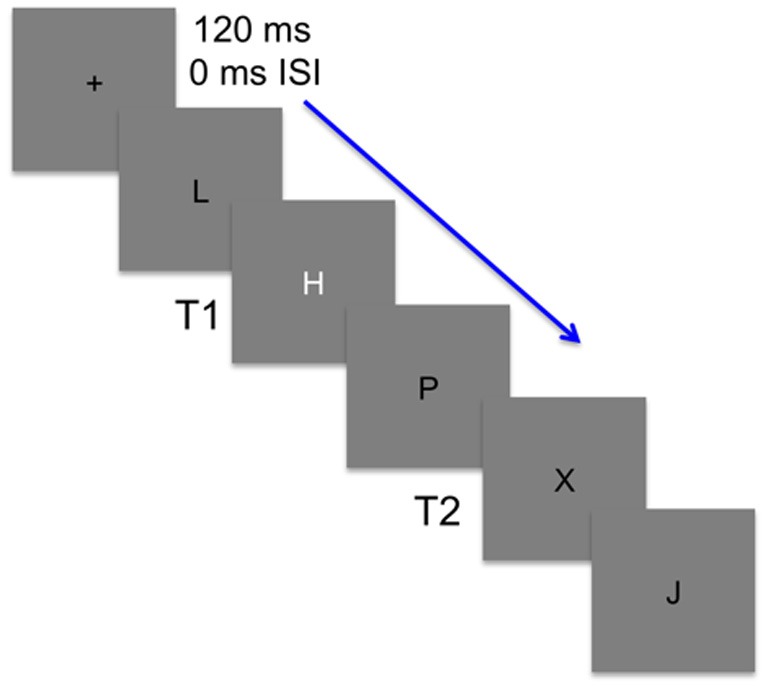
**An illustration of the stimuli used in the attentional blink task.** Stimuli were presented at a rate of 120 ms with no inter-stimulus interval (ISI). Participants were to first detect whether a white target (T1) was embedded among black distractors. T1 was either an H or S and on one-third of the trials was replaced by a black distractor. Participants then needed to detect a second target (T2) that randomly occurred 1–8 lags after T1 and was black like the distractors. T2 was present in every trial and was either an X or Y.

Participants were instructed to direct their attention to the center of the screen. Each trial began with the presentation of a small, white dot at center fixation that lasted 180 ms in duration. Letter stimuli succeeded the cue and the first task of the participants was to detect a white target letter presented among black letter distractors. The white target (T1) was either an H or S or did not occur at all. In every trial there was always a black X or Y target (T2) and participants were to also identify which target was presented. Manual responses to T1 and T2 were made after the RSVP of letters and were prompted by separate screens of instructions. For T1, participants were to press “H” on the keyboard if they saw H, “S” if they saw S, or “N” if no T1 occurred. For T2, participants were instructed to press “1” if they saw X and “2” if they saw Y (see Figure 1). Importance was placed on accuracy and participants were encouraged to guess on trials when they were unsure. Target accuracy was recorded using Eprime software; no reaction time was recorded or emphasized. Participants performed 5 blocks of 72 trials before and after cTBS stimulation.

### Theta burst stimulation procedure

Application of cTBS was performed with a MagPro ×100 stimulation unit (Medtronic, Minneapolis, MN, USA) using a Figure 8 coil (MCF-B65). For stimulation of the left cerebellar hemisphere the centre of the coil was placed 1 cm below and 3 cm to the left of the inion. For the right hemisphere the coil was placed 1 cm below and 3 cm to the right of the inion (Theoret et al., 2001). Stimulation intensity was set at 80% of active motor threshold (AMT) for the right first dorsal interosseous (FDI) muscle. To determine AMT, the stimulation coil was placed against the upper left surface of the participant's scalp at the optimal position for eliciting motor-evoked potentials (MEPs) from the contralateral FDI muscle. AMT was defined as the lowest stimulator output required to produce a MEP of >200 μV peak-to-peak for 5 out of 10 trials during a 10% maximum voluntary isometric contraction of the right FDI. For sham stimulation, the TMS unit was set to 6% of maximum output so that participants could hear the stimulus pulses; however the coil was oriented up and outward from the scalp over either the left or right cerebellar target. This was done to simulate stimulation in naïve participants. Stimulation settings consisted of 600 pulses delivered over 40 seconds, applied in a theta burst pattern consisting of three pulses at 50 Hz repeated at 5 Hz. This pattern replicated that used by Huang et al. (2005).

### Data analysis

To assess whether all three stimulation groups performed similarly pre-cTBS, T2 detection accuracies were submitted to analyses of variance (ANOVAs) in which lag (six positions) was a within-subject factor and group (left, right, and sham) was a between-subject variable. ANOVAs were also performed to test T1 detection accuracy across groups as well as to test T2 detection accuracy when it occurred in trials without the presentation of T1 (control condition). For T2 detection accuracy, only trials with a correct response for T1 were used for analysis. The same analyses were performed post-cTBS, including paired contrasts to test the specific *a priori* hypothesis that there would be poorer performance in T2 accuracy during short lags after cTBS for the right cerebellar hemisphere group compared to the left cerebellar hemisphere and sham group.

## Results

Analyses of the demographic data for the participants revealed no significant difference between groups on age [*F*_(2, 42)_ = 3.09, *p* = 0.06]. Means for the left, right, and sham group were 23.5 (SD = 3.34), 24.8 (SD = 3.82), and 21.8 (SD = 2.68), respectively.

### Pre-stimulation

#### Accuracy

**Figure 2:**
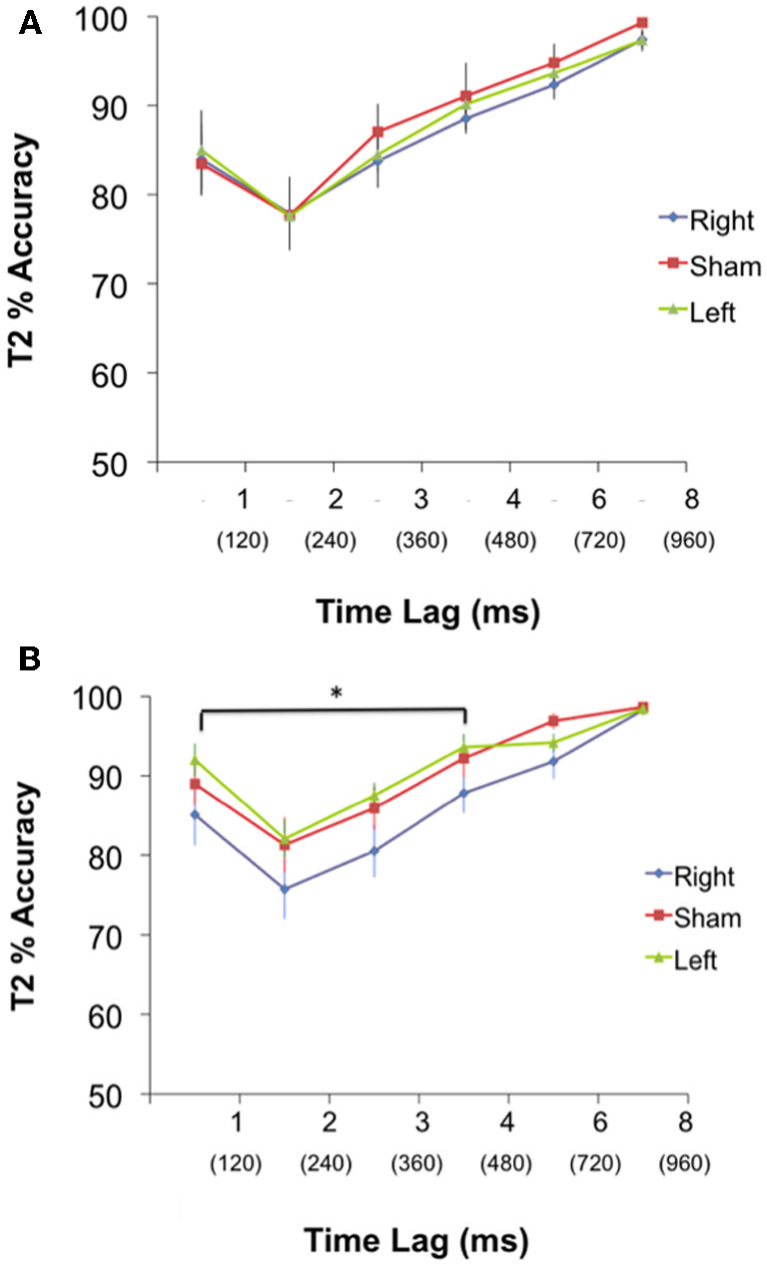
**(A)** PRE: Performance (Mean % accuracy ± S.E.M) in detecting T2 for the Left, Right, and Sham group during pre-cTBS condition. There was no significant difference in performance between groups at any lag. Time between each lag was 120 ms. **(B)** POST: Performance (Mean % accuracy ± S.E.M) in detecting T2 for the Left, Right and Sham group during post-cTBS condition. Paired contrasts reveal a significant difference between the right group and both the left and sham group for lags 1–4, ^*^p < 0.05. Time between each lag was 120 ms, and T2 at lags 1–4 occurred within 480 ms of T1.

***T2 detection (AB condition)***. A 3 group × 6 lag ANOVA of T2 accuracy was performed. The test revealed no significant interaction (*p* = 0.99) or main effect of group [*F*_(2, 42)_ = 0.56, *p* = 0.57], but a main effect of lag [*F*_(5, 39)_ = 23.58, *p* = 0.001] (Figure 2A). Thus, while all groups responded similarly to the position of T2 with respect to T1, there was no difference amongst the groups at each lag.

***T1 detection***. All groups were actively engaged in identifying T1 (99% for left, 99% for right, and 99% sham). There was no significant difference in T1 accuracy between groups (*p* = 0.64).

***T2 alone (control condition)***. Accuracy at detecting T2 is virtually unimpaired when it is not preceded by another target. There was no difference between groups in detecting T2 in the absence of T1 (94% for left, 94% for right, and 94% sham), (*p* = 0.99).

### Post-stimulation

#### Accuracy

***T2 detection (AB condition)***. A 3 group × 6 lag ANOVA revealed no significant interaction (*p* = 0.91) but a main effect of group [*F*_(2, 42)_ = 5.27, *p* = 0.006], and a main effect of lag [*F*_(5, 43)_ = 25.44, *p* = 0.001]. To probe at what lags the groups differed; a planned contrast was performed to test our *a priori* hypothesis that the right cerebellar group would have a greater AB magnitude during short lags compared to the left and sham group. Group means for the planned contrasts revealed a significant difference between the right and both the left and sham (*p* = 0.004) but no difference between left and sham (*p* = 0.38) during the short lags (Figure 2). There were no differences between the right and both left and sham groups (*p* = 0.15), or between the left and sham groups (*p* = 0.18) for the long lags. See Figure 2B.

***T1 detection***. cTBS had no effect on the accuracy of detecting T1. There was no significant difference in T1 accuracy between groups (*p* = 0.67). Group means were 99% for left, 99% for right, and 99% for sham.

***T2 alone (control condition)***. There was no difference in detecting T2 in the absence of T1 between groups (96% for left, 94% for right, and 95% sham), (*p* = 0.28).

## Discussion

The cerebellum is best known for its role in coordinating our movements to perform smooth and efficient actions. However, the cerebellum also modulates behavior outside the motor domain and is involved in rapid visual attention.

We found that the right posterior-lateral cerebellum is an essential node in AB performance. While there was no difference in performance across groups in the pre-cTBS condition, a main effect of group was found after stimulation. Post-cTBS, there was a larger magnitude of the AB in the short lags for the right cerebellar group compared to left and sham stimulation. This supports our main hypothesis, that the right cerebellum is recruited in the AB network when the temporal constraints of the AB task are high. Also, performance at detecting T1 or T2 alone was not influenced by cTBS, suggesting that the right cerebellum is not involved in the general detection of a target, and is specific to the accurate detection of a target stimulus when it occurs within half a second of another target stimulus. Thus, disrupting the posterior-lateral cerebellum in healthy participants provides evidence that parallels previous cerebellar patient data (Schweizer et al., 2007), and for the first time provides specificity to the contribution of the cerebellum to the AB. The role of the right posterior-lateral cerebellum in the AB task is not surprising, as other cognitive tasks that use language-based stimuli are associated with this area (Desmond et al., 1997; Richter et al., 2007; Schweizer et al., 2010).

There has been recent evidence that the contralateral connections between the cerebral cortex and cerebellum are functionally segregated (Schmahmann et al., 2009). Anterograde transneural virus tracers have identified projections from the dorsolateral prefrontal cortex (area 46) to the lateral cerebellar cortex (Crus II) that had no overlap with arm area projections from the primary motor cortex to cerebellar lobules IV–VI (Kelly and Strick, 2003). Thus, the role of the cerebellum is not restricted to motor coordination and may be involved in modulating function in the motor and cognitive domain alike. We have evidence that supports a role for the cerebellum in the AB, however, understanding its precise role remains elusive. Contributing to this is the fact that there are many interpretations of how the AB phenomenon occurs. Most common is the idea that the AB reflects the inefficiency of managing attentional resources, where if too many attentional resources are allocated to T1, it increases susceptibility to distractor interference and performance on T2 suffers if it is presented before consolidation of T1 can occur (Giesbrecht and Di Lollo, 1998). Based on this account, it is possible that the cerebellum is involved in the efficient allocation or coordination of attentional resources to T1, so that the likelihood of distractor interference is decreased and the opportunity for T2 detection is increased. However, according to Lavie's load theory (2005), if the perceptual load of a target is low, the likelihood of distractors disrupting performance is high. This is because less attentional resources are required to process the target, and more are left open and vulnerable to distractors. In the case of the AB, T1 is always quite salient and easily detected. Therefore, the increased AB magnitude at shorter lags may be a result of too few resources being attended to T1, and too many being susceptible to distractors, decreasing the prospect of T2 detection. The cerebellum may thus be recruited to efficiently modulate the attentional resources dedicated to T1 to readily detect T2. This would also account for why T2 accuracy is decreased after right cerebellar stimulation even at Lag 1, where no distractor occurs between the two targets. Regardless of how the cerebellum is involved in the AB, we speculate that the involvement of the cerebellum is driven by a left frontal-right cerebellar network, recruited during the early lags to rapidly detect both targets. More time is able to elapse between targets at later lags and therefore the demand for readily available attentional resources is decreased. Disruption to this same network decreased performance in a word generation task that required fast and efficient mental flexibility (Arasanz et al., 2012). Kornysheva and colleagues (2011) reported that rTMS to the ventral premotor cortex increased activity in the cerebellum particularly in subjects that showed the smallest reduction in performance during an auditory-motor timing task. Cerebellar activity served as a predictor of task accuracy, with highest activity in less impaired subjects. Thus, the cerebellum may be recruited when additional or a reorganization of resources is required.

This study also provides further support for the use of cTBS as a neuroimaging tool to explore the causal relationship between the cerebellum and cognitive functioning. While a somatotopic organization of a sensorimotor map within the cerebellum has previously been identified in animals (Snider and Eldred, 1951) and humans (Grodd et al., 2005), it has recently been proposed that this functional topography extends to higher-order brain areas (Stoodley and Schmahmann, 2010; Stoodley et al., 2012). Using cTBS, we targeted the posterior-lateral cerebellar cortex, which topographically corresponds to a cerebellar subregion involved in cognitive functioning. By transiently disrupting this focal area, cTBS can provide a cleaner, more precise functional map of the cerebellum. This technique has an advantage over fMRI, as the BOLD response is simply correlational and does not provide a causal relationship between brain and behavior. Continuous TBS also has an advantage over lesion studies, as it can provide local specificity; while the location of cerebellar damage can vary patient to patient.

The use of letters as stimuli during the AB task is commonly accepted in the literature and was specifically chosen for its localization in the right cerebellar hemisphere. However, to support our finding that the cerebellum's contribution to the AB is hemisphere specific, future studies using other stimulus features may be beneficial. A limitation to the design of this study is that when T2 occurred at lag 8, no other stimuli in the letter stream followed T2, leaving it unmasked and easier to detect (Vogel and Luck, 2002). This may have contributed to the high accuracy performance at the long lags, however at lag 8 T2 occurs 960 ms after T1, which is far outside the boundaries of the AB (Raymond et al., 1992). Another potential limitation to this study is the fact that it is strictly behavioral. Future studies are needed that combine TMS and EEG to elucidate how the cerebellum contributes to the AB network by comparing neural markers such as the P300, which is correlated to the AB phenomenon.

Based on our results, the network recruited for fast and efficient control of attentional resources during the AB involves the cerebellum. The role of the cerebellum in this network is hemisphere specific, localized to the right posterior-lateral cerebellar cortex. The goal of our future studies is to determine how the cerebellum contributes to the AB network.

### Conflict of interest statement

The authors declare that the research was conducted in the absence of any commercial or financial relationships that could be construed as a potential conflict of interest.
